# A Novel Redundant Validation IoT System for Affective Learning Based on Facial Expressions and Biological Signals

**DOI:** 10.3390/s22072773

**Published:** 2022-04-04

**Authors:** Antonio Costantino Marceddu, Luigi Pugliese, Jacopo Sini, Gustavo Ramirez Espinosa, Mohammadreza Amel Solouki, Pietro Chiavassa, Edoardo Giusto, Bartolomeo Montrucchio, Massimo Violante, Francesco De Pace

**Affiliations:** 1 Department of Control and Computer Engineering, Politecnico di Torino, 10129 Turin, Italy; antonio.marceddu@polito.it (A.C.M.); luigi.pugliese@polito.it (L.P.); ramirez.g@javeriana.edu.co (G.R.E.); mohammadreza.amelsolouki@polito.it (M.A.S.); pietro.chiavassa@polito.it (P.C.); edoardo.giusto@polito.it (E.G.); bartolomeo.montrucchio@polito.it (B.M.); massimo.violante@polito.it (M.V.); 2 Electronics Department, Engineering School, Pontificia Universidad Javeriana, Bogota 1301, Colombia; 3 Institute of Visual Computing and Human-Centered Technology, Vienna University of Technology (TU Wien), 1040 Vienna, Austria; francesco.pace@tuwien.ac.at

**Keywords:** facial expressions, image databases, neural networks, heart rate variability, physiological data, behavioral analysis

## Abstract

Teaching is an activity that requires understanding the class’s reaction to evaluate the teaching methodology effectiveness. This operation can be easy to achieve in small classrooms, while it may be challenging to do in classes of 50 or more students. This paper proposes a novel Internet of Things (IoT) system to aid teachers in their work based on the redundant use of non-invasive techniques such as facial expression recognition and physiological data analysis. Facial expression recognition is performed using a Convolutional Neural Network (CNN), while physiological data are obtained via Photoplethysmography (PPG). By recurring to Russel’s model, we grouped the most important Ekman’s facial expressions recognized by CNN into active and passive. Then, operations such as thresholding and windowing were performed to make it possible to compare and analyze the results from both sources. Using a window size of 100 samples, both sources have detected a level of attention of about 55.5% for the in-presence lectures tests. By comparing results coming from in-presence and pre-recorded remote lectures, it is possible to note that, thanks to validation with physiological data, facial expressions alone seem useful in determining students’ level of attention for in-presence lectures.

## 1. Introduction

The activity of teaching requires questioning oneself at every moment and guessing independently if the topics are being explained effectively or not. Such skills usually improve through experience by observing classroom reactions to different methodologies and lecture content. This operation can be simple in small classrooms, while it may be challenging with classes of 50 or more students. In addition to this, large classrooms usually encounter difficulties in terms of student–teacher interaction and the visibility and audibility of lessons.

The scope of this paper is to propose a novel Internet of Things (IoT) system based on non-invasive techniques and designed to aid teachers in getting feedback about the students’ level of attention. Facial expression recognition is used as primary source, while physiological data analysis, since it requires students to wear a smartwatch, is used as a validation tool when available.

To benchmark the proposed approach, 13 students were involved in two different measurement campaigns, performed in two different contexts: in-presence and remote lectures.

While the proposed methodology to perform facial expression recognition and physiological data analysis are well described in the literature, to the best of our knowledge, there are only a few proposals on how to analyze data from both sources to obtain a redundant system. We claim that, by merging data from both sources, it is possible to obtain a comprehensive image of the level of attention in a classroom.

Considering facial expression recognition, a Convolutional Neural Network (CNN) was used to recognize eight emotions which, alongside others, are currently accepted as common to all cultures: *anger*, *contempt*, *disgust*, *fear*, *happiness*, *neutrality*, *sadness* and *surprise*.

In contrast, physiological reactions were measured by Photoplethysmography (PPG).

At this point, both paths are post-processed to obtain a percentage of attention shown by the students during a specific time interval.

Privacy issues are also considered. The proposed methodology analyses only classroom data, avoiding collecting data of a specific user. Hence, the data are averaged before the computation of the level of attention.

The rest of the paper is organized as follows. Section 2 presents the state-of-the-art on facial expression recognition through neural networks and physiological data analysis. Section 3 describes the proposed approach, explaining the data analysis from both the sources and how it is possible to merge them. Section 4 presents the experimental results obtained from the two campaigns. Finally, Section 5 draws some conclusions and future directions.

## 2. Background

Human cognitive abilities have been widely studied from neurophysiological and psychological points of view. Different theories and pedagogical methodologies were derived to optimize cognitive processes of the human mind [1]. Since computers and electronic devices are increasingly used for in-presence and remote teaching activities, the students’ level of attention might be negatively affected by the possible distractions that these devices bring [2,3]. Research evidence shows a strong correlation between different cognitive processes and physiological responses, which among others, can be reflected in the heart rate and eye blinking. The observation of these phenomena, as a consequence, allows the cognitive state of a human being to be established [4].

These variables have been collected and analyzed to determine the attention and emotional factors that can affect the learning process. In [5], previous hypotheses proposed that the student’s attention decreases over time are refuted through physiological measurements. These are presented as a less subjective way to determine the level of engagement. In [6], an extensive review is carried out about the different neurophysiological variables used to determine the levels of attention, which can be classified into two different groups: the central nervous system (CNS) and the autonomic nervous system (ANS). With the former group, the variables are directly linked to the central nervous system, that is, the measurements require invasive techniques for the target student, limiting their comfort and deploying possibilities of these systems on a large scale. Illustrative examples can be found in [7,8], whose authors employ electroencephalography (EEG) to determine attention and learning levels. On the other hand, the latter group concerns the ANS signals, which are commonly represented and modeled using facial expressions, eye-based measurements, Heart Rate (HR), Blood Volume Pressure (BVP), and Skin Conductance (SC). These variables provide less invasive measurement for the students. The detection of attention and emotional factors is strongly related to ANS signals and it can be divided into two main approaches: facial expression and multi-neurophysiological signal detection.

### 2.1. Facial Expression Recognition

Techniques for emotion recognition from facial expressions have been studied for years.

In 1971, American Psychologist Paul Ekman and William Friesen published a list of the six primal emotions shared among all human groups, independently from their culture: anger, disgust, fear, happiness, sadness, and surprise [9]. These emotions were further expanded in other works by Ekman itself, Daniel Cordaro and other researchers [10,11]. In 1978, Ekman also introduced the Facial Action Coding System (FACS), which permits to classify the muscular movements of the human face [12]. It also identified 46 Action Units (AUs) that are responsible for facial movements. The combination of multiple actions and their respective intensities generates a large set of possible facial expressions. Only experienced annotators can classify facial expressions by using FACS with good accuracy.

In [13], the authors described the channels of information that are used to transmit emotions, recognizing facial expressions and vocal intonations as the main ones. They also divided the process of affect recognition from facial expressions into three stages: face detection, facial features extraction, and, finally, the description of the affective state.

While the goal of the first two stages is clearly defined, the latter is more complex due to the subjectivity of the task. According to them, the description of the emotional state must be identical to a human’s description of the same affective state. However, they remark that this interpretation can vary from person to person and culture to culture. For this reason, they reckon that affection recognition programs must be well-tailored to the context in which they are used.

The extraction of facial features is often performed trying to recognize AUs of FACS, rather than prototypic expressions of common emotions (happiness, anger, etc. …). This is because prototypic expressions occur rather infrequently, while emotions are often communicated by changes in discrete facial features [14]. In addition, AUs are studied by psychologists for the recognition of complex emotional states [15].

The study presented in [16] tried to improve some shortcomings of FACS, namely the lack of temporal and detailed spatial information, by introducing a new representation called FACS+. Similar techniques, based on optical flow, are also used in [17] for developing a time-aware method for facial expressions recognition.

The survey presented in [15], analyzes the most common techniques for affect recognition by decomposing their pipeline into fundamental components: face registration, representation, dimensionality reduction, and recognition.

The face can be initially registered either as a whole, a combination of parts or by the localization of fiducial points. It is then encoded in a spatial or spatio-temporal representation. Spatial representation encodes image sequences frame by frame and can be performed at different levels of abstraction.

Low abstraction levels representations are achieved with techniques such as Local Binary Pattern (LBP), Local Phase Quantization (LPQ), and Gabor Filters. In [18], a combination of Gabor Filters and LBPs is adopted. High-level representation, instead, tries to extract features that can be semantically interpretable.

For what concerns dimensionality reduction, it can be either pooling, feature selection, or feature reduction.

Finally, in the recognition phase, the output is provided as labels of emotions or facial actions, such as AUs. Common techniques are Hidden Markov Model (HMM), Support Vector Machine (SVM), Dynamic Bayesian Network (DBN), Relevance Vector Machine (RVM), and Conditional Random Field (CRF).

Another point of interest is the variety of datasets that are used for the training and validation of affect recognition techniques. The first distinction is made on how the dataset is labeled: some use FACS [19] while others just report prototypical expressions [20,21].

Some datasets contain posed expressions [19,20], sometimes performed by actors [21], while others are generated from the labeling of natural images. Moreover, they can also contain subtle [21], or exaggerated expressions [22,23]. Pictures can be generated ad hoc for emotion recognition purposes, or taken from the Internet and movies [24].

With the increased popularity of neural networks in the past years, there have been many attempts of using them to improve affect recognition tasks. In [18], the authors mixed a more standard pipeline, composed of traditional Gabor Filters and LBPs combined with Extreme Learning Machines (ELM), useful for real-time applications.

Deep Learning models are also used, but they have some limitations due to the limited number of available images [25]. For this reason, in [26] a Shallow CNN is preferred to recognize micro-expression with a limited number of training samples. In [25], instead, this problem is tackled by strongly driving the model toward the relevant facial areas by using domain knowledge.

In [27], a Deep Belief Network (DBN) is trained to perform the three principal phases of feature learning, feature selection, and classifier construction in a unified loopy framework. Instead, [28] introduced an identity-aware CNN to mitigate variations introduced by personal attributes and achieved better facial expressions recognition performance. In [29], an Artificial Neural Network (ANN) is trained with a database ensemble to make the model less sensible to image variability and increase the number of available samples. In [30], a Contractive Convolutional Network (CCNET) is used in order to obtain invariance to translations of the facial traits in the image.

### 2.2. Multi-Neurophysiological Signal Recognition

Another research approach consists of measuring different ANS neurophysiological signals providing reliable emotion and attention measurement. In [31], the authors proposed a cyber-physical social system by exploiting multiple sensors and cameras in conjunction with a quiz creator to keep track of the learning process of students. They did so by applying reinforcement learning techniques to the data, allowing the teacher to increase the engagement of students.

The reinforcement learning algorithm uses the students’ heartbeats, eye blinks, facial expressions to recommend the next learning activity depending on their learning state.

In [32], the authors developed an automated mechanism for the classification of student engagement during a writing task.

They used computer vision techniques to extract three sets of features from videos: HR, facial feature, and Local Binary Patterns in Three Orthogonal Planes (LBP-TOP). They build engagement-detection models for each of the features, plus two fusion models, using all the features and a subset of those.

Their implementation suffered from some limitation with respect to the facial feature extraction and heart rate estimation, both carried out via video processing.

In [33], the authors proposed a model of e-learning which provides learning activities according to the condition of mental or emotional state. The model under this information makes an automatic selection of the lessons. It takes as input biophysical variables such as HR, SC, and BVP to determine the affective state.

Another rising methodology to detect physiological and biological characteristics is the use of PPG technology. It is a simple and low-cost optical method that can detect blood volume changes in the microvascular bed of tissue beneath the skin due to the circulatory system’s pulsatile nature.

The PPG is a non-invasive electro-optical technique to measure beats associated with changes in the bulk of the bloodstream in the circumferential of the human body [34].

Alternating Current (AC) component counts as the pulsatile component of the PPG waveform and usually its fundamental frequency depends on heartbeat, which is generally around 1 Hz. AC and Direct Current (DC) can be extracted for subsequent pulse wave analysis through appropriate electronic filtering and amplification. Few optoelectronic components are required to create the simple structure of PPG technology: a light source to illuminate the tissue (e.g., skin) and a photodetector to measure the minor variations in light intensity associated with perfusion changes catchment volume.

The factors that affect the reproducibility of the PPG technique include the method of probe attachment to tissue, probe tissue interface pressure, pulse amplifier, bandwidth, minimization of movement artifact, subject posture and relaxation, breathing, wakefulness, room temperature, and acclimatization. Semiconductor technology has been developed in LED, photodiodes, and phototransistors. This improved size, sensitivity, portability, reliability, and reproducibility of the PPG probe design.

PPG is widely used in clinical physiological monitoring of parameters such as blood oxygen saturation, blood pressure, heart rate, heart rate variability [35]. The first advantage is that it permits to measure the physiological parameters without the need for electrodes, movable technology for primary care, and community-based clinical settings. Then, it uses simple, low-cost, and compact semiconductor devices. Finally, it is an advanced computer-based pulse wave analysis technique. The basic form of PPG technology requires only a few optoelectronic components: a light source to illuminate the tissue (e.g., skin) and a photodetector to measure the small variations in light intensity associated with perfusion changes catchment volume.

In the following, Heart Rate, Blood Pressure (BP), and Heart Rate Variability (HRV) will be discussed.

One of the vital physiological parameters analyzed in the clinical setting is Heart rate (HR). A source of HR information can be measured from the AC component of the PPG pulse, which is synchronous with the heartbeat. Using simple digital filtering and zero-crossing, the reliability of HR detection is improved so that HR and respiratory components can be separated from the PPG technique.

Blood pressure has also been well investigated in vascular disease studies. Blood pressure is calculated using an algorithm based on the pulse arrival time and compared with conventional blood pressure.

The physiological control of HR and BP is vital in maintaining BP homeostasis and is often characterized by BaroReflex Sensitivity (BRS, unit ms/mmHg). BRS can be attenuated in cardiovascular disease. Its non-invasive assessment is possible using beat-to-beat peripheral arterial BP waveforms, measured using finger-pressure cuff/PPG technology [35]. In [36], authors showed that Pulse Transit Time (PTT) at the finger and ECG RR interval is distinctly modulated by paced breathing (rate 0.1 Hz). Here, the incremental PTT and RR interval values of ECG on a beat-to-beat basis using an overlapping waveform morphology technique and their variability were summarized using simple statistics.

In the cardiovascular system, this nonstationary balance fluctuates between intervals of consecutive heartbeats and HRV.

The HRV is a set of measures that are utilized for the ANS action estimation. It can be expressed as PPG power spectrum feature (LF and HF), or Beat-to-Beat (BB) interval property. The BB features are usually utilized in the emotion field, and some of them are defined as [37]:

- SDNN, that is the Standard Deviation of BB intervals.
- SDSD, that is the Standard Deviation of Successive BB interval Differences.
- RMSSD, that is the Root Mean Square of Successive BB interval Differences.
- pNN50, that is the percentage of adjacent BB intervals that differ from each other by more than 50 ms.

At this time, the design and development of wearable biosensors have gathered significant attention in academics and marketing due to their possible applications in human health monitoring and personalized medicine. In general, Wearable BioSensors (WBSs) are portable electronic devices that integrate sensors into/or with the human body in different forms and shapes such as watches, gloves, clothing, and implants [38]. WBSs use smart portable devices to analyze and record live sensing data of human biological parameters such as blood pressure, heart rate, and temperature that possess significant values in healthcare applications [39]. Moreover, the conventional PPG sensors must be firmly attached to the skin to get a good, high-quality signal. Wearable sensors with accompanying measuring functions have solved and detected various notable problems in the health, medical, and sport fields. In our research, authors recurred to smartwatches which are popularly categorized as wrist-worn wearable devices, to record signals as heart rate.

### 2.3. Internet of Things for Affective Learning

We are living in the era of the Internet of Things [40], or Internet of Everything, with billions of devices connected to the internet and carrying out a particular activity. Such devices are employed both for personal use or company/society purposes, for instance, fitness tracking or environmental monitoring [41]. It is possible to exploit these devices to produce interesting information that can be analyzed for decision-making or lifestyle adaptations. The landscape of these sensing/actuating devices is so vast that they face several limitations concerning the scalability and integration of systems. This is why conceptions such as the Social Internet of Things [42] and the related regulations have been proposed [43,44,45].

The application of IoT technologies to educational fields becomes a support technology and a research trend in improving the regular classroom. It permits to facilitate and adapt the learning processes by collecting and analyzing data extracted from the educational contexts [46]. Our aim is to exploit the IoT domain potential to improve feedback information collection of a teacher willing to engage the students as much as possible.

Indeed, this new domain merging has already been explored by other researchers. In [47], the authors exploit a WiFi-enabled Radio Frequency Identification (RFID) reader to assist teachers in automatically recording attendance to lessons and students’ behavior. They combine this with the use of Quick Response (QR) codes to quickly access course materials and provide the capability of real-time interactive responses to stimulate participation. The implementation of this system reportedly increased the attendance rate from 85% to 98%.

In another paper [48], the authors develop a voice assistant application for Google Home which can interact with teachers and students depending on textbook contexts. Their model uses Machine Learning algorithms to recognize user sentences and learn new ones. They present the results of two case studies carried out in Bangladesh.

More specifically, in affective learning [49], the authors proposed an IoT-based framework to detect human emotions applied to special education, wherewith the help of wearable devices are used to detect heart rate, movement, and environmental data such as temperature and humidity. They use the data gathered to monitor students with attention deficit hyperactivity disorder and provide affective feedback to the educators. An alternative IoT Framework is proposed in [50]; in this research, the authors develop an emotion recognition system that analyzes datasets from diverse ANS and CNS physiological signals. Afterward, a transmission analysis was made to ensure real-time communication to the IoT hub to identify emotions. The authors’ goal is to provide a solution for remote learning and healthcare support in the context of the confinement generated by the COVID-19 emergency.

## 3. Methodology

The proposed methodology is based on a redundant system, with two data sources. To improve the clarity of the work carried out, this section was divided into three main parts:

1. Facial expression recognition through neural networks Section 3.1;
2. Physiological data analysis Section 3.2;
3. Merging of the information Section 3.3.

### 3.1. Facial Expression Recognition through Neural Networks

As seen in Section 2.1, facial expression recognition task can be accomplished through the use of artificial intelligence systems. Through a shrewd training phase, they can approximate a function capable of recognizing the people’s facial expressions portrayed in an image or a video. Neural networks are one of the techniques offered by artificial intelligence, and they are increasingly used mainly for the high precision they are able to achieve. Among all possible neural networks, the proposed approach is based on the neural network presented and trained in [29]. Interested readers can refer to this paper for additional details.

Training a machine learning system that can discriminate between a limited number of classes requires the presence of labeled examples. For example, if we take a dining chair, a rocking chair, and an office chair, they are still chairs even if they have differences in shape, size, color, and more. The same can be said for dining tables, living room tables, office tables, and others. For this reason, the use of categorization supergroups and the visualization of the multitude of characteristics that an object can have is extremely important for a machine learning system. The labeling process must, therefore, be carried out painstakingly, reducing the number of errors to what is humanly possible [51].

#### 3.1.1. Training a Neural Network through Quality Image Database

In these years, many high-quality facial expression databases were released. In [29], several neural networks based on [25] CNN were trained through the Keras library [52] by using a combination of different facial expression databases, called Databases Ensembles (DEs). The use of multiple databases adds strength to the trained system, as they can:

- have a different photographic quality;
- have different backgrounds;
- portray multiple human traits;
- increase the size of the train, validation, and test dataset.

This allows for the creation of artificial intelligence systems able to generalize more and be reliable even in conditions that diverge more from those of training. As seen in Section 2.1, since facial expressions can be either posed or spontaneous, two different DEs were created:

- One, called “Ensemble 1”, consisting only of image databases containing people with posed and spontaneous facial expressions:
  - Extended Cohn-Kanade Database (CK+) [19,53];
  - FACES Database [54];
  - Facial Expression Recognition 2013 Database (FER2013) [22] plus FER+ annotations [23];
  - Japanese Female Facial Expression (JAFFE) [20];
  - Multimedia Understanding Group Database (MUG) [55];
  - Radboud Faces Database (RaFD) [56].
  - Static Facial Expressions in the Wild (SFEW 2.0) [24].
- Another, called “Ensemble 2”, consisting of image databases containing only posed facial expressions:
  - CK+;
  - FACES Database;
  - JAFFE;
  - MUG;
  - RaFD.

For reasons which will be explained in the following lines, the authors have again resorted to the neural network obtained by using the “Ensemble 1” DE. It was composed of 7 different databases for a total of 43,993 photos, divided into 8 different facial expressions, each representing an emotion:

- Anger;
- Contempt;
- Disgust;
- Fear;
- Happiness;
- Neutrality;
- Sadness;
- Surprise.

Its creation was made easier by using facial expression database Classifier (FEDC) [57], a software which provide support for DEs creation. Images were preprocessed according to the following steps:

- Grayscale conversion;
- Resize to 48 × 48 pixels;
- Face cropping;
- Z-score normalization;
- 90–10% random split for train and test datasets.

To increase the system accuracy and obtain the validation dataset, 9-fold cross-validation was performed to the first subset to achieve a final split of 80%-10%-10%. During each training epoch, the following data augmentation operations were resorted to:

- Random horizontal flip;
- Brightness range among 50% and 100%;
- Shear range among ±2.5%;
- Random rotation among ±2.5 degrees;
- Zoom transformation interval among ±2.5%;
- Width and height shift range among 2.5%.

**Figure 1:**
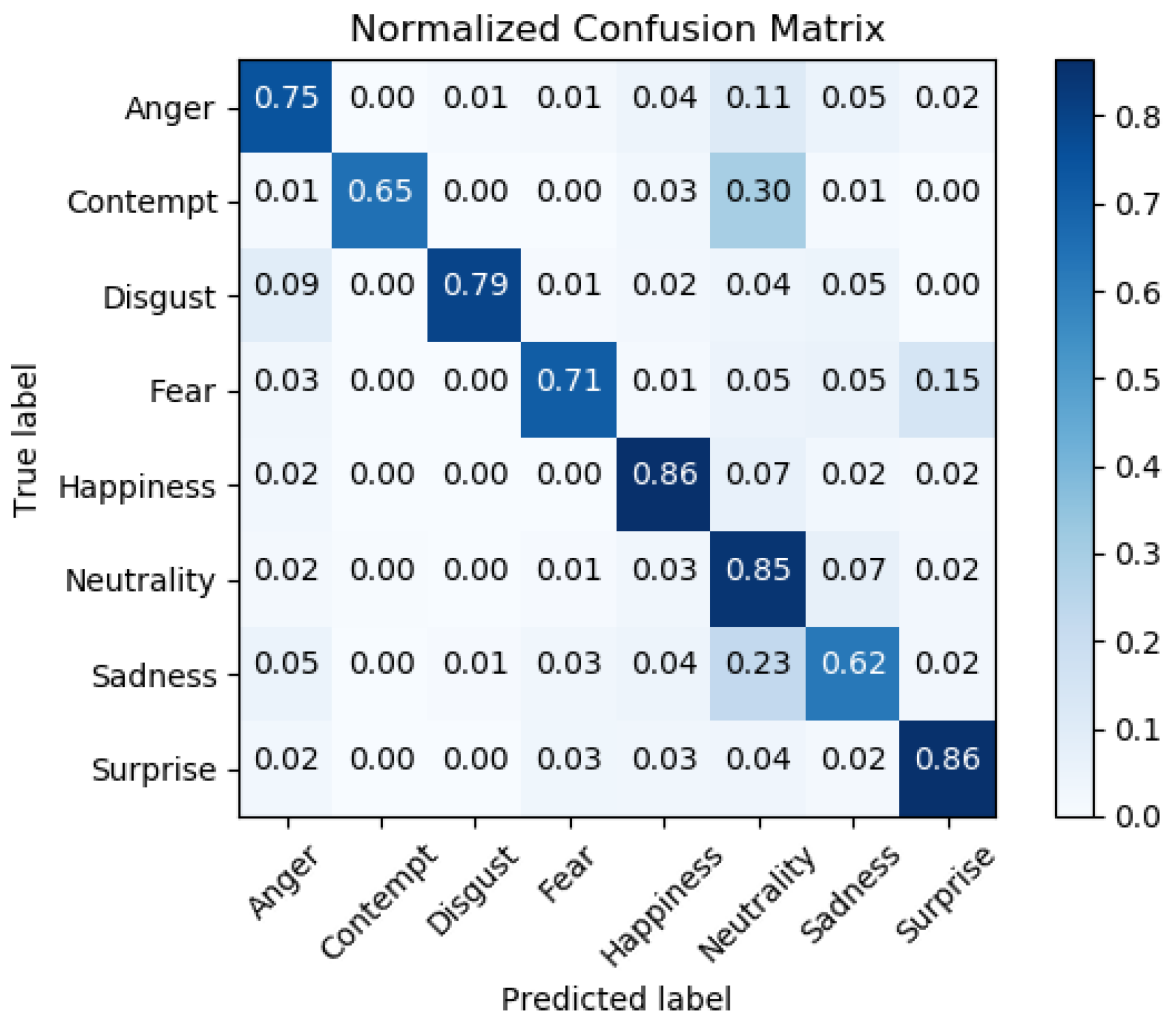
Normalized confusion matrix of the neural network described in [25], trained by using the “Ensemble 1” dataset [29].

The best fold combination for the CNN described in [25], obtained using “Ensemble 1” as train, validation, and test dataset and using the settings described above, achieved a test accuracy of 80.38%. The respective confusion Matrix is displayed in Figure 1.

This is quite low compared to the accuracies obtained using only CK+ (92.5%) and “Ensemble 2” (96.78%) datasets. The problems of the latter databases are that:

- they contain few images. This reduces the ability of the neural network to generalize and so to work well in the real case;
- they are composed of images taken in a photographic studio. This implies less variability than the real use case;
- only posed facial expressions are represented. This creates a bias to the more frequently used spontaneous ones, which are more natural but with less inter-class variability [29].

Due to these reasons, the neural network obtained using “Ensemble 1” as train, validation, and test dataset, even if it presents the worst quantitative metrics, was considered reliable for the considered facial expression recognition problem and thus employed for the proposed approach.

#### 3.1.2. Facial Expressions Prediction and Postprocessing

Facial expressions prediction was achieved through the use of Emotion Detector (ED) software [51,58]. It was developed by recurring to:

- Java;
- Apache Maven;
- OpenCV [59];
- DeepLearning4J (dl4j) [60].

It can recognize the facial expressions of a single person by taking the camera, a video, or an image as input. Since the neural network was built to analyze single frames, a sampling rate must be selected if a video is provided. Regardless of it, the software:

1. Displays the current image or frame;
2. Performs face detection using Haar Cascades [61];
3. Updates the interface by drawing the detected face in an ad hoc box. Otherwise, “face not found” warning is shown;
4. Performs facial expression recognition using the neural network discussed in Section 3.1;
5. Updates the graph by showing the newly obtained result;
6. Optionally, it saves the result as a screenshot or in a Comma-Separated Values (CSV) format file.

For this project, it was decided to improve ED by adding the possibility to choose between performing face detection via Haar Cascade Classifiers or with a pre-trained Deep Neural Network (DNN), which is able to perform the same task with greater precision. It consists of a Single Shot MultiBox Detector (SSD) [62] based on ResNet-10 architecture [63] trained with the Caffe framework [64] which is able to detect faces on 300 × 300 pictures. The latter setting was used for facial expressions analysis. Videos collected from the in-presence and remote lesson were analyzed using ED by capturing 2 samples per second. The results were then exported to CSV format for further analysis.

To align facial expressions predictions obtained through CNN with physiological data, they were grouped into two moods, by following a Russell’s-like classification [65]:

- active:
  - Anger;
  - Fear;
  - Happiness;
  - Surprise;
- Passive:
  - Contempt;
  - Disgust;
  - Sadness.

The active ones corresponds to the high arousal moods, while the passive correspond to the low arousal ones. The pleasure-displeasure characteristics are not considered for the sake of this work.

Facial expressions depicting neutrality have been excluded from this classification since it is not classifiable as either active or passive.

Then, an activation level has been determined by counting the active moods readouts into a specific time interval (in this case, 100 s) and normalizing it to $Ws_{NN}=300$ (i.e., 300 represents an interval with only active moods, while 0 an interval with only passive moods).

It is convenient to apply a binary classification on the activation level by comparing it with a Threshold ($T_{fe}$), corresponding to $Ws_{NN}/3$, obtaining two different levels stored in an Attention Array from facial expressions ($AA_{fe}$). This binary array contains a value for each time interval (corresponding to the number of active moods in the last 100 samples): if the grade of attention is higher than $T_{fe}$, that window is labeled as “Attention”, otherwise as “Distraction”.

### 3.2. Physiological Data Analysis

This section describes the Attention Detection algorithm based on physiological reaction. The data have been acquired using a set of commercial smartwatches (Garmin Venu Sq) that transfer data via BLE (Bluetooth Low Energy) to a set of smartphones, with a sampling rate of 1 Hz. The collected information is saved in a text file.

The acquired datasets are composed of HR and HRV. The algorithm has been entirely developed using MATLAB. Before executing it, a two levels calibration phase focused on thresholds estimation for the emotional stages detection, is performed on each patient:

1. Based on their information, which are age, gender, weight, and height;
2. From their initial condition estimation.

The algorithm can work live during the lecture to perform a behavioral analysis. This entails observing the HR and the HRV for a duration of *N* samples, defined in the following as Window Size (WS). From this observation window, only some of the presented emotional stages are utilized: SDNN, RMSS, and SDSD, which were described in Section 2.2. By sliding the window, with a specific initial delay, it is possible to generate an output every second.

By comparing the punctual value of the three parameters with the one coming from the calibration phase, it is possible to increase the Grade of Attention (GA). Similarly to the classification of the attention levels, GA is compared with a Threshold ($T_{p}$), calculated as:

$$T_{p}=\frac{3}{2}\cdot \mathrm{WS}$$

where 3 is the number of the calculated emotional stages. In this way, two levels stored in an Attention Array from physiological reactions ($AA_{p}$) were obtained. As for the activation levels obtained from neural network classification, if the grade of attention is higher than $T_{p}$, that window is labeled as “Attention”, otherwise as “Distraction”.

### 3.3. Merging Physiological and Facial Expressions Data

The process to obtain $AA_{fe}$ and $AA_{p}$ is needed to allow comparing data from these very different sources. Then, it is possible to apply the windowing algorithm.

The purpose of the study is not to analyze the attention of a single student but to obtain aggregated results. The reasons why to not consider a single student are the following:

- Make system results not affected by the interest of a single student on the lecture topic;
- The same student, on different days, can show different interest in lectures due to personal reasons (i.e., drowsiness and moods);
- The CNN performing facial expression recognition can be affected by somatic features which diverge too much from the training data.

An important parameter to be determined is the WS. The output of the windowing algorithm represents the number of “Attention” labels in the sliding window. This window ends at the considered time and starts WS samples before. Since people’s facial expressions change rapidly, neural network output can have fast variations. To smooth the results, a 5 min attention counter filter is applied to obtain the averaged activity level, defined as Attention Behavior (AB). Due to this reason, the algorithm will effectively start to work after 5 min. For coherency, the same operation is also applied to physiological data.

**Figure 2:**
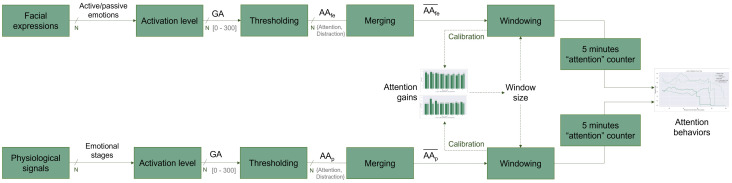
The workflow of the proposed analysis approach. The dashed arrows represent the WS calibration feedback.

A step-by-step summary of the proposed approach is shown in Figure 2.

### 3.4. Comparison of Methodology with Other Solutions

This project aims to use physiological data to validate those from facial expressions. This could lead to the creation of a system capable of resorting only to facial expressions. The advantages of such a system are that:

- It is less invasive.
- Could be applied to both in-presence and remote lectures.
- It is less expensive as it requires only webcams or similar devices.

These are non-trivial since the previously mentioned systems recurred to a multimodal acquisition methodology not easily implementable in everyday use.

For example, compared to [33], the experiments were conducted using consumer-grade devices, having a lower cost. The new technologies then made it possible to obtain similar results using fewer parameters. In particular, while in [33] BVP, SC and HR were used, only HRV and HR were used in this work. Another difference is that the facial expression recognition system can effectively handle multiple people. It is based on two CNNs, one capable of extracting the face and the other of extracting the facial expression. For this reason, this system can be used without problems even during in-presence lectures and not only in remote ones, in which there is individual fruition.

Another example is that in [32] the tests were conducted using a similar number of students but in a different context (writing activity). The face is traced using a Kinect, while HRV and HR are extracted by using ECG. The problem with such technologies is that they are not easily applicable in a realistic environment. Moreover, engagement is estimated using LPB-TOP and a machine learning tool that uses custom classifiers.

## 4. Experimental Results

As reported in Section 2 [2,3], online classrooms may reduce the level of attention with respect to the in-presence one due to the distraction induced by the electronic devices, also the one used to attend the lecture itself.

On the contrary, when they are physically in the same room, students/professor interaction is usually higher [66].

Due to these reasons, it was decided to test the level of attention in both lecture modes. A total of 13 people were enrolled, 4 females and 9 males, aged from 21 to 35.

The first campaign consisted of in-person lectures. The following data have been collected:

- Physiological data, thanks to commercial smartwatches;
- Facial expressions, thanks to visible-wavelength cameras.

More details about this first campaign are described in Section 4.1.

The second campaign consisted of remote lectures. The following data have been collected:

- Physiological data, thanks to smartwatches;
- Facial expressions, thanks to visible-wavelength cameras (at the purpose, the volunteers were asked to record their facial expressions using their webcams or mobile phones cameras);
- Reaction times, thanks to an application designed for this purpose.

More details about this second campaign are described in Section 4.2.

### 4.1. In-Person Lectures

Two lectures of 1 h length were held in-presence, each one with 4 participants, for a total of 8 volunteers.

Two experimenters conducted the in-person lectures. The first assumed the role of the teacher, while the second was in charge of programming the teacher’s wristwatch to alert him/her with a vibration at set times not known by the teacher.

When an alarm elapsed, the teacher requested the volunteers to perform a simple action (for example, touch their shoulder, nose, or head) and asked them if they were paying attention to the explanation.

The reaction times were measured by resorting to the video recording of the lecture, starting from the moment when the teacher asks to perform the actions, to the time at which the actions were carried out.

In order to carry on a fair comparison, only the first lecture group was aware of the request.

### 4.2. Remote Lectures

Remote lectures, which became more common after the SARS-CoV-2 pandemic of 2020, were experimented.

To perform these lectures, 6 volunteers attended a prerecorded lecture of about 50 min and recorded their facial expressions using a personal device of their choice. They were also provided with smartwatches to monitor their physiological reactions. Moreover, since no lecturer was present, an application was used to randomly ask the students if they were paying attention to the lecture or not.

#### Application to Measure the Response Times

The application, called Reaction Time Tool, was developed using Microsoft.NET 6 framework and C# language. The Human–Machine Interface (HMI) has been designed with the Windows Presentation Foundation (WPF) environment.

Its HMI is composed of two main windows: the first one is shown at the program startup and asks the volunteers where they want to save the log file, then it shows a blinking hourglass to assure the user that the application is working correctly.

At predefined times, not known by the volunteers, a message window appears, asking if they are paying attention to the lecture. Depending on its grade of attention, the user can answer “Yes” or “No”. It is not possible to close this window without answering.

The application emits a sound to catch the user’s attention when the message window appears. This sound has been chosen to be different from any of the sounds commonly emitted by the usual message windows of the operating systems. Whenever the window is shown, the application saves a timestamp of the event into the log file. A second event is saved when it is closed, alongside the answer provided by the volunteers. Moreover, to simplify the data analysis, the application computes the Δ time between the showing of the window and the user answer. Of course, this application does not run on a real-time system but an ordinary personal computer.

Furthermore, in this campaign, one group of 4 people knows about these message windows in advance, while the other group of 2 does not.

It is expected that, from the second time on, users would recognize them faster with respect to the first time.

Resuming, the purposes of this application are twofold:

1. It asks the students if they are paying attention to the lecture;
2. It records the reaction time of the student.

The source code of this application is available for interested readers on GitHub under the MIT license [67].

As a comparison between the attention level obtained from both sources in the in-presence and remote lectures and the self-assessment of the volunteers, we obtained the following results.

For the in-presence lecture, when the students were asked if they were paying attention or not, we got that 5 of them answered yes every time they were asked, while 3 always answered no. These answers are approximately 40%.

Each volunteer was asked the same question 5 times using the Reaction Time Tool for the remote lectures, obtaining 20 affirmative answers and 5 negatives. However, 2 of the 20 affirmative answers have been given with an excessive reaction time; for this reason, we considered them as false answers. With 18 yes and 7 no, we got an attention level of 72% measured with a window size of 10 min.

This result appears a bit far from the measurements obtained with the proposed approach (around 52%), but it is not possible to associate a statistical relevance due to the small number of volunteers.

### 4.3. Window Size Determination

The first problem was to determine the WS for the windowing algorithm. In the literature, there is little information about the behavioral analysis on the emotional stage parameters, so it has been determined empirically by choosing the value that minimizes the readout differences when the windowing algorithm is applied on $\bar{AA_{fe}}$ and $\bar{AA_{p}}$.

**Figure 3:**
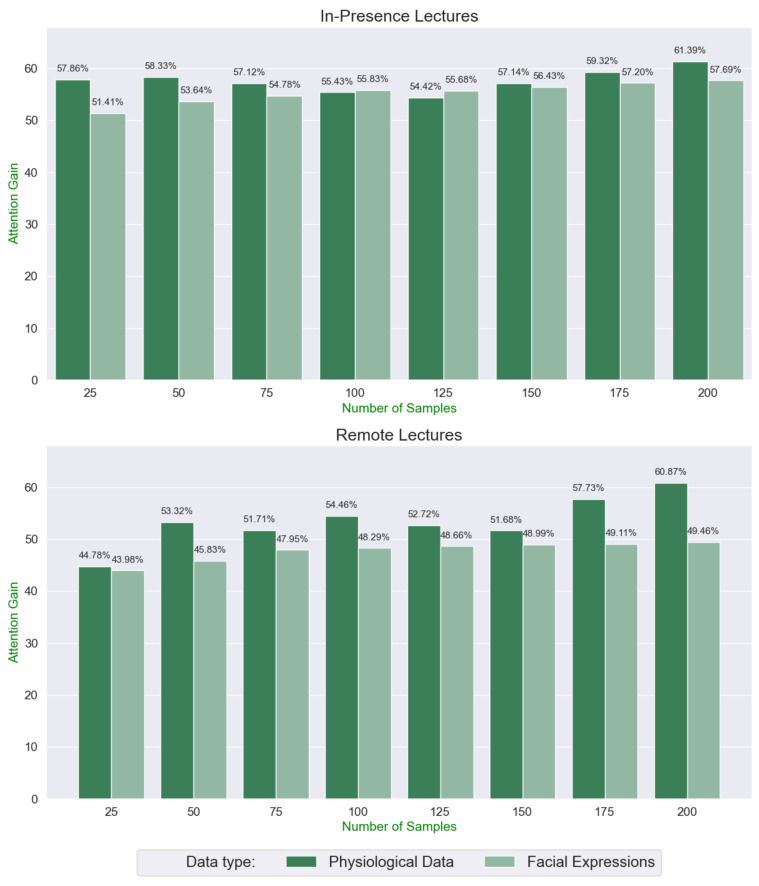
Comparison of the *AG*s obtained from the $\bar{AA_{p}}$ and $\bar{AA_{fe}}$ during the in-presence and remote lectures with different windows sizes.

Figure 3 shows the redundancy between the two systems. The Attention Gain (*AG*) represents the percentage of “Attention” states detected from the raw (without applying the 5 min attention counter) ABs. The graphs highlight that the best results for the in-presence lectures were obtained for 100 samples WS. It has been decided to use the *AG*s from the in-presence lectures since the Neural Network performed better in this case. In the remote lectures, it is possible to observe a strong presence of “Neutrality” outcomes, making the results from this source less predictive w.r.t. the ones from the Physiological Data.

Taking into account 100 samples of both sources, the Root Mean Square Error (RMSE) can be calculated as follows:

$$\mathrm{RMSE}=\sqrt{\frac{{\left(\hat{AG}-AG_{p}\right)}^{2}+{\left(\hat{AG}-AG_{fe}\right)}^{2}}{2}}$$

Having $AG_{p}$ and $AG_{fe}$ as their respective *AG*s from the physiological data and facial expressions and $\hat{AG}=\left(AG_{p}+AG_{fe}\right)/2$ as the mean between $AG_{p}$ and $AG_{fe}$, it was obtained that RMSE${}_{presence}=0.02$ and RMSE${}_{remote}=4.76$.

### 4.4. Attention Behavior Analysis

**Figure 4:**
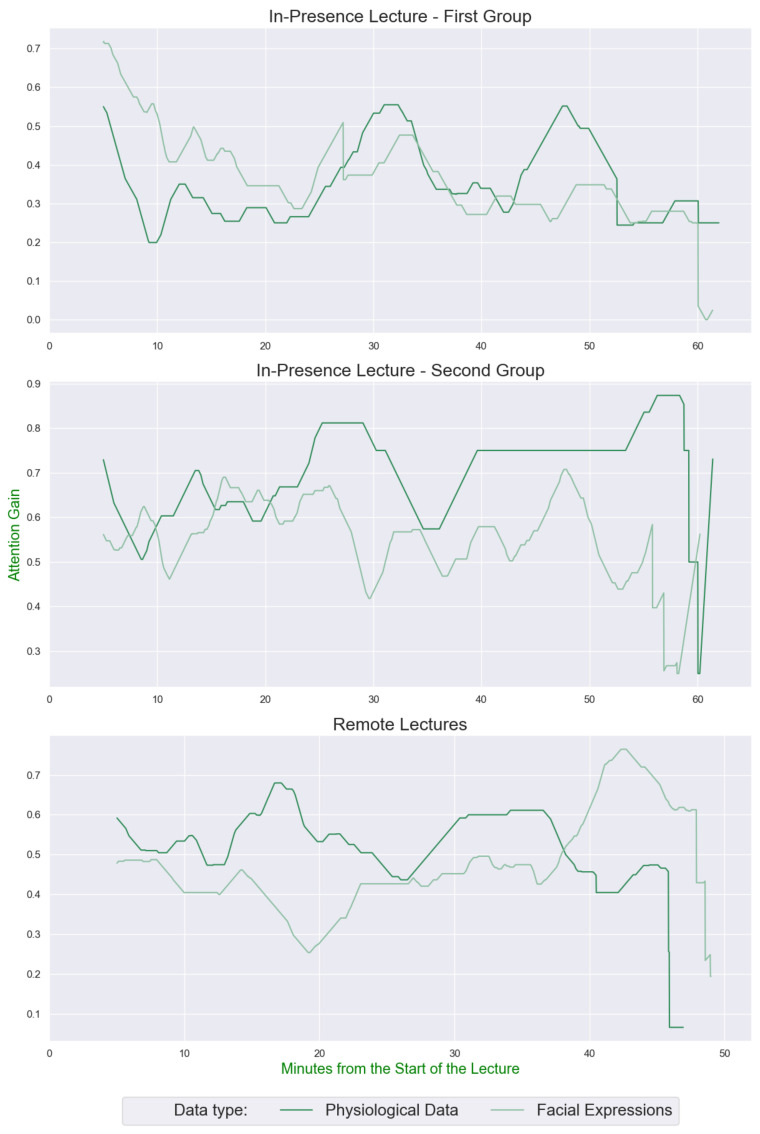
ABs of the in-presence and remote lectures obtained from $\bar{AA_{p}}$ and $\bar{AA_{fe}}$ with a WS of 100 samples and applying the 5 min attention counters every second.

As the WS has been determined, it is possible to compute the AB. To analyze the students’ collective AB, it is necessary to filter the windowing algorithm results. The results shown in Figure 4 have been obtained by counting the number of “Attention” states detected in the previous 5 min of the $AA_{i}$.

The reaction times have been collected but not used in this work because a correlation between attention level and reaction times has not been found in the literature. In any case, this data collection is available, and it has been decided to describe it in this paper due to the perturbation on the student attention levels due to the attention checks, which can be relevant to allow replication of these experiments.

As written before, data were available only after 5 min from the start of the lecture. This delay does not affect the AB analysis and, since lectures usually last more than 1 h, it is acceptable for a real-world working application.

### 4.5. Results Discussion

The results provide interesting insights regarding the students’ attention behaviors in the in-presence and remote lectures. As evidenced in Section 4, it is immediately visible the different levels of attention between the lecture types. Moreover, the ABs calculated from both sources agree with each other.

Figure 4 shows the average attention status of the in-presence and remote groups, respectively. Referring to it, an attention gap between the two groups is immediately visible. The average values indicate that the second group paid more attention than the first one. It is reasonable to assume that the second group:

- knew that actions could be required during the lecture and this may have induced a greater state of attention due to their happening;
- may have felt a greater interest in the topic of the lecture than the first.

Due to the passive type of lesson, neutrality facial expressions prevail in the remote lecturer. For this reason, Physiological Data can be quite useful. The peak of attention in the in-presence lectures happening after around 20 min for the second group, and 30 min for the first one, can be explained by a more interesting topic to the audience with respect to the lecture average. Moreover, in one of the in-presence lecture a student interacted a lot with the teacher, while in the other, after 25 min, there was the request to touch their heads.

Furthermore, the flat results of the remote lectures can be explained in the same way: the topic is more technical (how to design an IT component) with respect to the in-presence one that is more informative (how the airplanes can fly), so it is expectable that the engagement level has fewer variations.

Finally, it is possible to notice how the results obtained in this work are similar to those obtained in the two works deepened in Section 3.4.

## 5. Conclusions

This paper focuses on the redundancy of two different systems to be used when ground truth is not available. As the use case, it has been decided to measure students’ attention level in two different setups: in-presence and remote lectures.

Two technical contributions are present: one based on facial expression recognition through CNN and a deterministic algorithm developed to analyze physiological reactions. These two methodologies are well established, so a novel redundant approach to merge data from these two sources has been proposed. The CNN produces emotions classification, while the physiological algorithm outputs “Attention” or “Distraction” labels. For this reason, much effort was put into producing data in the novel form of attention arrays, which make it possible to compare the readouts together. The proposed IoT system could be helpful to tell teachers the level of attention in their classrooms. It can highlight the lessons’ moments through attention feedback, aiding them to improve the quality of the lessons or increase the interaction with students.

It is important to remark that such a system cannot produce data about the attention level of a single student, so it is crucial never to provide data obtained before the merging operation. This aggregation also solves privacy issues.

As mentioned in Section 4.4, a strong presence of Neutrality outcomes, as happened in the experimental campaign presented in this paper, makes the results from the Neural Network less reliable with respect to the one achieved by the Physiological Data analysis.

An improvement of Neural Network performances could allow mitigating this issue.

Following the road to redundancy, it is possible to add other data sources. An example of a possible extension could be the introduction of a blink counter and a controller for determining eye gaze direction.

## Abbreviations

The following abbreviations are used in this manuscript:

$AA_{fe}$	Attention Array obtained from a single volunteer from facial expressions
$AA_{b}$	Attention Array obtained from a single volunteer from physiological reactions
$\bar{AA_{fe}}$ and $\bar{AA_{b}}$	Attention Array obtained from all volunteers after the merging operation
AB	Attention Behavior
AC	Alternating Current
AG	Attention Gain
ANN	Artificial Neural Network
ANS	Autonomic Nervous System
AU	Action Unit
BB	Beat-to-Beat
BLE	Bluetooth Low Energy
BVP	Blood Volume Pressure
CCNET	Contractive Convolutional Network
CNN	Convolutional Neural Network
CNS	Central Nervous System
CRF	Conditional Random Field
CK+	Extended Cohn-Kanade Database
DBN	Deep Belief Network or Dynamic Bayesian Network
DC	Direct Current
DE	Database Ensemble
Dl4j	DeepLearning4J
DNN	Deep Neural Network
ED	Emotion Detector
EEG	Electroencephalography
ELM	Extreme Learning Machine
FACS	Facial Action Coding System
FEDC	Facial Expression Database Classifier
FER2013	Facial Expression Recognition 2013
HCI	Human–Computer Interaction
HF	High Frequency
HMI	Human–Machine Interface
HMM	Hidden Markov Model
HR	Heart Rate
HRV	Heart Rate Variability
IoT	Internet of Things
JAFFE	Japanese Female Facial Expression
LBP	Local Binary Patterns
LBP-TOP	Local Binary Pattern on Three Orthogonal Planes
LED	Light-Emitting Diodes
LF	Low Frequency
LPQ	Local Phase Quantization
MUG	Multimedia Understanding Group
pNN50	Percentage of adjacent BB intervals that differ from each other
	by more than 50 ms
PPG	Photoplethysmogram
PTT	Pulse Transit Time
QR	Quick Response
RaFD	Radboud Faces Database
RFID	Radio Frequency IDentification
RMSE	Root Mean Square Error
RMSS	Root Mean Square of successive BB interval differences
RR	Respiration Rate
RVM	Relevance Vector Machine
SARS-CoV-2	Severe Acute Respiratory Syndrome Coronavirus 2
SC	Skin Conductance
SDNN	Standard Deviation of BB intervals
SDSD	Standard Deviation of successive BB interval differences
SFEW 2.0	Static Facial Expression in the Wild 2.0
SSD	Single Shot MultiBox Detector
SVM	Support Vector Machine
WBSs	Wearable BioSensors
WPF	Windows Presentation Foundation
WS	Window Size
